# Intention to use a health information platform in supportive housing for people with disabilities: An application of the UTAUT model

**DOI:** 10.1371/journal.pone.0332072

**Published:** 2025-10-09

**Authors:** Bohye Kim, Hye Jin Nam, Haesun Lee, Hannah Park, Ju Young Yoon

**Affiliations:** 1 College of Nursing, Seoul National University, Seoul, South Korea; 2 Center for Human-Caring Nurse Leaders for the Future by Brain Korea 21 (BK 21) Four Project, College of Nursing, Seoul National University, Seoul, South Korea; 3 Gangdong Public Health Center, Seoul, South Korea; 4 Department of Nursing, Dongguk University WISE, Gyeongju, South Korea; 5 Research Institute of Nursing Science, Seoul National University, Seoul, South Korea; Swiss Paraplegic Research, SWITZERLAND

## Abstract

Staff in supportive housing for people with disabilities face challenges in delivering effective health management, highlighting the need for a health information communication and management platform (referred to as “the platform”). This study aimed to identify factors influencing staff’s intention to use the platform by applying the Unified Theory of Acceptance and Use of Technology (UTAUT) model, with the addition of eHealth literacy. A self-report questionnaire survey was conducted from August 1 to September 30, 2024. Data analysis included descriptive statistics, correlations, and multiple regression. The 200 participants consisted of 70 men (35.0%) and 130 women (65.0%). Significant positive correlations were found between intention to use the platform and all UTAUT variables (p < .001). Multiple regression analysis showed that voluntariness (β = 0.58, p < .001), performance expectancy (β = 0.20, p < .001), facilitating conditions (β = 0.12, p = .040), and eHealth literacy (β = 0.10, p = .042) significantly predicted staff’s intention to use the platform, explaining 67.7% of the variance. Voluntariness, performance expectancy, facilitating conditions, and eHealth literacy significantly influence staff’s intention to adopt the platform, offering key insights for developing a user-friendly platform for supportive housing for people with disabilities.

## Introduction

People with disabilities are increasingly moving into community living environments, prompting discussions on how to ensure their health rights. There is also greater interest in the health management capabilities of caregiving staff working in supportive housing for people with disabilities. Supportive housing integrates caregiving and housing functions to facilitate independent living for individuals with disabilities within the community. It also provides personalized residential support and assistance services, reflecting the individual needs and characteristics of residents based on the principle of self-determination of people with disabilities [1]. Specific services include assistance with household tasks, support for employment and participation in community activities to promote independent living, health lifestyle support, medication management, and assistance with hospital and clinic utilization to maintain and improve the health of residents [2]. However, staff in supportive housing—primarily social workers, formal caregivers, and personal support workers—face challenges in effectively addressing the complex health and medical needs of people with disabilities.

Although supportive housing aims to promote independent living, many limitations have been reported in terms of health management due to the insufficient health knowledge and skills of the staff [3]. Accordingly, researchers and practitioners have recommended introducing systems to address the health management challenges caregiving staff face [3]. Specifically, real-time health monitoring and information sharing through information and communication technology (ICT) have been suggested approaches to alleviate the burden on staff and systematically manage the health of people with disabilities [3]. ICT refers to technologies that facilitate the collection, storage, processing, transmission, and exchange of information through digital and analog tools, such as the internet and electronic devices, based on users’ needs and objectives [4]. The use of ICT enables caregiving staff to easily access and utilize care-related information [5]. ICT has also been shown to reduce caregiving-related stress and anxiety by fostering emotional support through social networks, and ultimately enhancing self-efficacy [6]. In addition, providing appropriate care based on ongoing integrated information has the effect of reducing negative health outcomes, such as hospital admissions [7,8]. However, studies have highlighted the various challenges of using ICT including user characteristics, such as age or digital literacy [9], and the significant time required to effectively use these systems [6].

The COVID-19 pandemic has significantly highlighted the potential and advantages of ICT in care settings for people with disabilities, leading to an accelerated integration of ICT into service delivery. However, there remains a critical need for further investigation and empirical research to understand and optimize the use of ICT in these care settings [10,11]. While previous studies have emphasized the importance of ICT in health management for people with disabilities, their focus has largely remained on medical institutions [12]. Specifically, a digital integrated healthcare service platform utilizes Internet of Things (IoT) devices and patient-generated health data to enhance communication among people with disabilities, caregivers, and healthcare professionals, demonstrating the potential impact of ICT-based healthcare systems to improve the efficiency of health management for people with disabilities [12]. Furthermore, prior research has developed a web-based application to provide customized health management for people with disabilities in the community setting [13]. However, in community-based settings such as supportive housing for people with disabilities, the development and implementation of such technologies are very limited. Supportive housing has a structure that makes it difficult to share systematic and consistent health information due to shift-based work and involvement of diverse care staff [3,14]. Therefore, it is critical to develop a health information communication and management platform (hereafter “the platform”) using ICT that can comprehensively support the communication and management of health information in supportive housing [3]. Such a platform is expected to enhance communication among the staff, strengthen the health management capabilities of people with disabilities, and ultimately improve the quality of health management within a supportive housing environment.

Since the pandemic, practitioners working in care settings for people with disabilities have actively used ICT mainly for information sharing and communication across residential and community settings [15,16]. Furthermore, the use of ICT is expected to expand to include tools for treatment, rehabilitation, and monitoring [15]. With the increasing use of ICT, it is essential to develop tailored strategies that address the specific needs of these settings.

Therefore, it is important not only to develop such platforms but also to understand practitioners’ acceptance of new technologies [17]. Most existing studies focus on healthcare professionals in clinical settings [18], while little is known about acceptance factors in community-based, non-medical environments. In the context of care settings for people with disabilities, practitioners’ perceptions and acceptance of ICT are key to successful implementation [16,19]. However, current ICT in these settings is often designed without adequately considering the user perspective, which limits their applicability in practice [20]. Thus, the aim of the study is to offer a comprehensive understanding of key factors that influence the staff’s intention to use the platform in supportive housing, based on the Unified Theory of Acceptance and Use of Technology (UTAUT) model. This understanding will provide a theoretical and practical foundation for guiding the development of user-centered platforms.

### Theoretical background: the UTAUT model

This study adopted the UTAUT model to explain the staff’s intention to use the platform in supportive housing for people with disabilities. The UTAUT model was developed to provide an integrated framework for understanding technology acceptance by combining eight theories/models: social cognitive theory (SCT), theory of reasoned action (TRA), motivational model (MM), theory of planned behavior (TPB), technology acceptance model (TAM), model of PC utilization (MPCU), innovation diffusion theory (IDT), and combined TAM and TPB [21]. The model includes four constructs that influence behavioral intention and use behavior: performance expectancy, effort expectancy, social influence, and facilitating conditions [21]. It explains that behavioral intention is influenced by the first three constructs (i.e., performance expectancy, effort expectancy, and social influence), while use behavior is directly influenced by facilitating conditions and behavioral intention. Additionally, gender, age, experience, and voluntariness of use serve as moderating variables that affect the relationships between the four constructs, behavioral intention, and use behavior [21]. It has been widely adopted and has consistently demonstrated strong reliability and validity for a wide range of technology acceptance in various groups [18].

Since the platform was still in development at the time of this study, we focused on the intention to use rather than the actual use behavior based on the framework of UTAUT model. In addition to performance expectancy, effort expectancy, social influence, and facilitating conditions that have been known to affect behavioral intention, voluntariness of use has also been identified as a significant predictor of behavioral intention [22,23] and thus was included as factors in this study. Furthermore, recent research has increasingly recognized eHealth literacy as an important factor influencing individuals’ acceptance of health technologies [24]. Accordingly, this study extends the UTAUT model by incorporating eHealth literacy as an additional variable. This reflects that the ability to understand and utilize information can have a significant impact on the actual acceptance of health-related technologies such as the platform.

Performance expectancy refers to the extent to which an individual believes that using the system will lead to improvements in job performance [21]. It is one of the strongest constructs influencing users’ intention to adopt new technology in organizational contexts. [25]. Users’ perception of the usefulness and benefits of new technologies has consistently been proven to significantly influence their behavioral intention and actual adoption [26–28]. It has been demonstrated that positive performance outcomes of technology use in healthcare influence attitudes toward its use [18,29,30]. Currently, the staff in supportive housing encounter numerous challenges in the health management of their residents [3]. Therefore, the implementation of ICT-based platforms is expected to improve their work efficiency and performance outcomes, which may consequently enhance positive perceptions of the platform and ultimately influence the intention to use it.

Effort expectancy refers to the degree of ease associated with using the system [21]. Many previous studies in healthcare have shown that effort expectancy was a significant predictor of intention to use healthcare technologies [18,27–29,31]. However, some studies have reported that effort expectancy does not significantly influence behavioral intention [30,32,33]. In the context of technology for the health management of people with disabilities, perceived ease of use is important to caregivers [34]. Although the staff may vary in their familiarity and proficiency with technology, platforms that require less effort to use are more likely to be accepted.

Social influence refers to the degree to which users believe that others who are important to them think they should use the system [21]. According to previous studies, it has been reported that individuals are influenced by others in their social networks, such as family, friends, and close colleagues when adopting new technologies [28,30,35]. Therefore, it is expected that when the staff receive positive information about the platform from others in their social network, their intention to use the platform will be positively influenced.

Facilitating conditions refer to the degree to which users believe that organizational and technical resources and support are available to assist with system usage [21]. Organizational and technical infrastructure are essential components for ensuring users’ intention to use technology. Prior studies on behavioral intention toward technology highlight the impact of facilitating conditions on users’ intention to adopt it [27,28,30,32,33]. A high level of support environment makes users perceive that the technology is easier to use, which has a positive effect on the intention to use the technology [28]. Hence, the staff’s perception that sufficient support and resources are available for use of the platform may positively influence their intention to adopt it.

Voluntariness of use refers to the degree to which users perceive that they have free will in adopting the system [21]. Voluntariness of use was treated as a moderating variable in the original UTAUT model, but some studies have treated it as an independent variable [22,23]. These studies have indicated that voluntariness significantly influences users’ intention to use technology [22,23,36]. Mandatory technology use within healthcare settings may generate user resistance, leading to negative outcomes [37]. Therefore, when the staff perceive that their use of the platform is voluntary, it is expected to have a positive effect on their intention to use it.

eHealth literacy is an important factor in adopting new technology in healthcare setting. eHealth literacy is a multidimensional concept that encompasses not only the ability to search for health-related information from electronic sources but also the capacity to comprehend, evaluate, and apply the information to address health issues [38]. With the increasing adoption of digital health technologies, eHealth literacy plays a critical role in the acceptance and use of digital health information management technologies [39]. Studies have shown that individuals with higher eHealth literacy are more likely to adopt and effectively utilize health information technology [40] and telemedicine [41]. Thus, eHealth literacy could be a key factor in enabling the staff to effectively utilize health management platforms.

**Fig 1:**
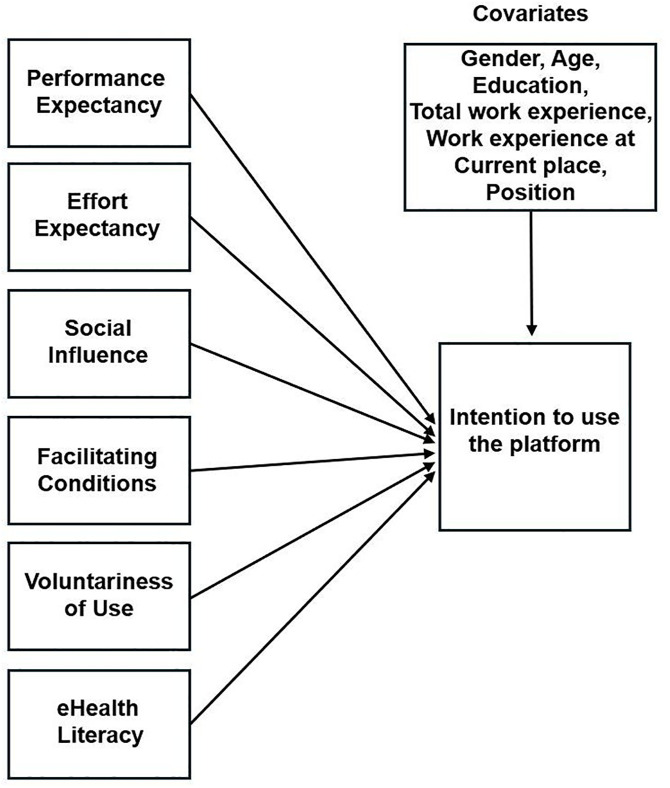
Conceptual framework of this study.

The UTAUT model has been widely applied in the healthcare field and has proven effective in predicting behavioral intention and actual usage behavior [16,18,42–44]. However, its application in the context of disability care, particularly in community-based supportive housing settings, remains limited. To address this gap, this study investigates the factors influencing the intention to use the platform among the staff in supportive housing in South Korea. Specifically, it aims to analyze the impact of the key UTAUT constructs (performance expectancy, effort expectancy, social influence, facilitating conditions, voluntariness of use) and eHealth literacy on intention to use the platform. This study contributes to the field of residential care for people with disabilities by extending the theoretical framework for technology adoption and providing practical implications for its effective implementation. Fig 1 shows the conceptual framework of this study. (Fig 1).

## Materials and methods

### Study design

We used a cross-sectional quantitative design with a paper-based survey to identify the factors influencing the intention to use the platform among staff in supportive housing.

### Participants

The participants were selected from staff working at nine supportive housing units in S City, South Korea. Recruitment documents were distributed at these facilities, and the survey was administered only to those who expressed interest in participating through the contact information provided in the document. The inclusion criteria for study participants were as follows: (1) at least three months of work experience in a supportive housing environment, (2) the ability to read and understand Korean and communicate effectively, and (3) voluntary agreement to participate.

### Instruments

#### General characteristics.

The general characteristics of the participants included gender, age, education level, total work experience caring for the elderly or people with disabilities, work experience at the current place, position, and the number of people with disabilities currently caring for.

#### eHealth literacy.

eHealth literacy was measured using the eHealth Literacy Scale (eHEALS) developed by Norman and Skinner [38], and the Korean version of the scale (KeHEALS), which was culturally adapted by Chang et al. [45]. The scale consists of 10 items. The first two items assessing participants’ interest in using internet health information, so these two items were not included in the scoring. The remaining eight items (items 3–10) measure participants’ eHealth literacy and were scored using a 5-point Likert scale. Items cover topics such as knowledge, judgment ability, and the use and evaluation of health information [38]. A higher score indicates a higher level of eHealth literacy, with scores above 26 indicating a high level and below 25 indicating a low level of eHealth literacy [46–48]. Cronbach’s α for the tool developed by Norman and Skinner [38] was 0.88. For the KeHEALS [45], it was 0.89, and for this study, Cronbach’s α was 0.93.

#### Key constructs of the UTAUT model and intention to use the platform.

The instruments used to measure the key constructs of the UTAUT model were adopted from validated items in prior studies [21,49], with minor modifications made to suit the platform for supportive housing.

1. Performance expectancy

Performance expectancy regarding the platform refers to the extent to which an individual believes that using the platform will improve job performance, and it consists of four items [49]. Each item is rated on a 5-point Likert scale, with higher scores indicating more positive performance expectancy. The Cronbach’s α for the original instrument was 0.90 [49], and in this study, it was 0.94.

2. Effort expectancy

Effort expectancy regarding the platform refers to the degree to which an individual believes that using the platform requires little effort, and it consists of four items [49]. Each item is rated on a 5-point Likert scale, with higher scores indicating more positive effort expectancy. The original instrument had a Cronbach’s α of 0.88 [49], and in this study, it was 0.96.

3. Social influence

Social influence regarding the platform refers to the degree to which an individual believes that important people around them think they should use the platform, and it consists of four items [49]. Each item is rated on a 5-point Likert scale, with higher scores indicating more positive social influence. The Cronbach’s α for the original instrument was reported as 0.85 [49], and in this study, it was 0.90.

4. Facilitating conditions

Facilitating conditions regarding the platform refer to the extent to which an individual believes that necessary organizational and technical infrastructure is available to support the use of the platform, and it consists of four items [49]. Each item is rated on a 5-point Likert scale, with higher scores indicating more positive facilitating conditions. The Cronbach’s α for the original instrument was 0.88 [49], and in this study, it was 0.89.

5. Voluntariness of use

Voluntariness of use of the platform refers to the degree to which an individual perceives that they have freedom in adopting the platform, and it consists of four items [49]. Each item is rated on a 5-point Likert scale, with higher scores indicating a more positive perception of voluntariness of use. The internal consistency of the original instrument was not reported [49], but in this study, the Cronbach’s α was 0.93.

6. Intention to use

Intention to use the platform refers to the behavioral intention to use the platform in the future, and it consists of three items [49]. Each item is rated on a 5-point Likert scale, with higher scores indicating a more positive intention to use the platform. The Cronbach’s α for the original instrument was 0.93 [49], and in this study, it was 0.96.

### Data collection

Data collection was conducted from August 1, 2024, to September 30, 2024, with only individuals who expressed interest in participating. All participants were provided with a written informed consent form along with the questionnaires. The survey consisted of 42 items and took approximately 20 minutes to complete. It provided information on the platform’s features and the functions it would offer, ensuring that participants had a basic understanding of the platform. A total of 207 participants initially expressed interest in the study. However, the final sample included 200 participants after excluding two individuals who did not meet the inclusion criteria due to insufficient work experience, four who withdrew from the survey, and one who participated multiple times. The required minimum sample size of 184 was calculated using the G*Power (version 3.1.9.7), based on a significance level of 0.05, an effect size of 0.15, statistical power of 0.95, and 12 predictors in the linear multiple regression analysis. This study’s sample size met these criteria, ensuring sufficient power to detect significant effects.

### Data analysis

The data collected in this study were analyzed using the SPSS/WIN 26.0 program. The general characteristics of the participants and the study variables were calculated using frequencies, percentages, means, and standard deviations. Independent t-tests and one-way ANOVA were used to compare differences in intention to use the platform based on the general characteristics and eHealth literacy of the participants. The correlations between performance expectancy, effort expectancy, social influence, facilitating conditions, voluntariness of use, and intention to use the platform were analyzed using Pearson correlation. Multiple regression analysis was conducted to examine the factors influencing intention to use the platform, including performance expectancy, effort expectancy, social influence, facilitating conditions, voluntariness, and eHealth literacy. Based on previous studies [50,51], general characteristics were set as covariates in the analysis including gender, age, education level, total work experience caring for elderly or people with disabilities, current work experience, and position. All statistical tests were two-tailed, and the level of significance was 5%. Missing data in the study variables were handled using the multiple imputation (MI) method [52].

### Ethical considerations

This study was approved by the Institutional Review Board of Seoul National University (IRB No. 2408/001–016). Written informed consent was obtained from all participants prior to data collection. Participants were informed that their involvement in the study, as well as their decision to withdraw at any point, would not result in any negative consequences. It was clearly stated that the data would be used solely for this research and stored securely in a coded format.

## Results

Table 1 shows the descriptive statistics and correlations for participants’ characteristics and intention to use the platform. A total of 200 participants were included in the study, consisting of 70 males (35.0%) and 130 females (65.0%), with a mean age of 56.24 years (SD = 11.18). The education levels of participants were as follows: middle school or lower (10.0%), high school (37.0%), 2-year college (13.0%), and university or higher (40.0%). The average total work experience of caring for elderly or people with disabilities was 65.70 months (SD = 59.87), and the average work experience at the current workplace was 35.08 months (SD = 25.92). In terms of job positions, 157 (78.5%) were categorized as personal support workers or coaches, while 43 (21.5%) held managerial positions (e.g., coordinator, team leader, or center director). In terms of the current workload, 54% of participants were caring for one individual with disabilities, while 16.5% were caring for two, and 14.0% for five or more. Regarding eHealth literacy, 165 participants (82.5%) were categorized as having high eHealth literacy, while 35 participants (17.5%) were in the low eHealth literacy group.

**Table 1:** Descriptive characteristics of study participants (N=200).

Characteristics	Categories	n or M	% or SD	F or t or r	*p*
Gender	Male	70	35.0	−0.20	.841
	Female	130	65.0		
Age (yr)		56.24	±11.18	−0.06	.433
		(range: 26–79)			
Education	≤ Middle school	20	10.0	2.46	.064
	High school	74	37.0		
	College	26	13.0		
	≥ University	80	40.0		
Total work experience caring for elderly or people with disabilities (mo)		65.70	±59.87	0.04	.546
		(range: 5–480)			
Work experience at current place (mo)		35.08	±25.92	−0.01	.865
		(range: 4–236)			
Position	Personal support worker or Coach	157	78.5	−1.55	.123
	Manager (coordinator, team leader, center director)	43	21.5		
Number of people with disabilities currently caring for	1	108	54.0	2.09	.084
	2	33	16.5		
	3	16	8.0		
	4	15	7.5		
	≥5	28	14.0		
		(range: 1–13)		0.16	.029
eHealth literacy	Low level	35	17.5	−4.24	<.001
	High level	165	82.5		
Performance expectancy		3.85	±0.71	0.64	<.001
		(range: 1–5)			
Effort expectancy		3.58	±0.83	0.53	<.001
		(range: 1–5)			
Social influence		3.52	±0.72	0.59	<.001
		(range: 1–5)			
Facilitating conditions		3.65	±0.68	0.59	<.001
		(range: 1–5)			
Voluntariness of use		3.71	±0.73	0.79	<.001
		(range: 1–5)			
Intention to use the platform		3.80	±0.72		
		(range: 1–5)			

mo = month; yr = year; M = mean; SD = standard deviations

Among participants’ characteristics, only eHealth literacy showed a significant difference in intention to use the platform (t = −4.24, p < .001). Additionally, a positive correlation was observed between the number of people with disabilities currently being cared for and intention to use the platform (r = 0.16, p = .029).

Descriptive statistics for the UTAUT variables revealed the following mean values: intention to use the platform (M = 3.80, SD = 0.72), performance expectancy (M = 3.85, SD = 0.71), effort expectancy (M = 3.58, SD = 0.83), social influence (M = 3.52, SD = 0.72), facilitating conditions (M = 3.65, SD = 0.68), and voluntariness of use (M = 3.71, SD = 0.73). Correlation analysis revealed significant positive relationships between intention to use the platform and all UTAUT variables (all p < .001). Voluntariness of use (r = 0.79) and performance expectancy (r = 0.64) showed the most significant correlations among the variables.

To analyze the factors influencing intention to use the platform, multiple regression analysis was conducted (Table 2). The regression model explained approximately 67.7% of the variance in intention to use the platform (adjusted R² = 0.677). Additionally, the variance inflation factors (VIF) were all below 10, ranging from 1.09 to 2.50, indicating no issues with multicollinearity. The Durbin-Watson statistic was 2.02, confirming that the residuals met the assumption of independence. The analysis revealed that voluntariness of use (β = 0.58, p < .001), performance expectancy (β = 0.20, p < .001), facilitating conditions (β = 0.12, p = .040), and eHealth literacy (β = 0.10, p = .042) significantly influenced intention to use the platform. Thus, higher levels of voluntariness of use, performance expectancy, and facilitating conditions, as well as being in the high eHealth literacy group, were associated with a greater intention to use the platform.

**Table 2:** Factors associated with intention to use the platform (N = 200).

Variables		B	SE	β	*t*	*p*
Gender	Female (ref. Male)	0.03	0.09	0.01	0.29	.775
Age		0.01	0.01	0.07	1.08	.282
Education	≥ College (ref. ≤ High school)	0.02	0.10	0.01	0.21	.833
Total work experience		0.001	0.001	0.05	1.08	.280
Work experience at current place		−0.001	0.002	−0.03	−0.63	.532
Position	Personal support worker or Coach (ref. Manager)	−0.11	0.15	−0.04	−0.72	.470
Performance expectancy		0.20	0.06	0.20	3.57	<.001
Effort expectancy		−0.06	0.06	−0.06	−0.98	.327
Social influence		0.07	0.06	0.07	1.24	.217
Facilitating conditions		0.12	0.06	0.12	2.07	.040
Voluntariness of use		0.58	0.06	0.58	9.09	<.001
eHealth literacy	High level (ref. Low level)	0.25	0.12	0.10	2.04	.042

R² = 0.696, adjR² = 0.677, Durbin-Watson = 2.022

B = Unstandardized regression coefficient; SE = Standard error; β = Standardized regression coefficient; ref. = Reference category.

## Discussion

The long-term health management of people with disabilities living in supportive housing is one of the key challenges. The complexity and diversity of residents’ health issues place a considerable burden on staff, emphasizing the need for a health information communication and management platform to support them [3]. This study is the first in South Korea to consider the introduction of such a platform and to examine the factors that influence users’ intention to adopt it, representing an important first step in this field. To this end, this study applied the UTAUT model to identify key factors that affect the intention to use the platform by the staff in supportive housing for people with disabilities. The findings revealed that voluntariness of use, performance expectancy, facilitating conditions, and eHealth literacy significantly influenced the intention to use the platform. These results highlight the need to promote voluntary participation, emphasize the platform’s potential to improve job performance, and ensure a supportive environment for its effective use. In particular, individuals with higher eHealth literacy showed a stronger intention to use the platform than those with lower eHealth literacy, emphasizing the essential role of eHealth literacy in platform adoption.

In this study, the mean intention to use the platform among the staff in supportive housing was 3.80, indicating a higher tendency compared to previous studies [26,53]. Although previous studies were different in terms of research subjects and environment, our results were higher. In particular, our results were higher in the intention to use eHealth interventions by health professionals in rehabilitation facilities (mean score: 2.47) [26] and the intention to use smart technology by long-term care staff in nursing homes (mean score: 3.33) [53]. This finding suggests a strong potential for adopting the platform in supportive housing environments. Supportive housing has been introduced in South Korea to promote independent living within the community as part of the deinstitutionalization policy for people with disabilities. However, much of the discussion has focused on housing and social participation, with comparatively limited attention and research on health management [54]. Although supportive housing accommodates residents with various chronic illnesses and complex health needs, most staff are professionals in the field of social welfare and often lack specialized knowledge and skills related to health management [3]. As a result, there are significant challenges in effectively addressing the complex health conditions of residents with disabilities, underscoring both the importance of health management and the need for the implementation of a systematic and professional health management system in these settings [3]. The high intention to use observed in this study reflects the urgent need for a health management system within supportive housing. In light of this, developing the platform from the early stages with consideration of the key factors influencing the intention of supportive housing staff to use it may lead to successful implementation.

This study found that voluntariness of use significantly influenced the intention to use the platform in supportive housing, which is consistent with prior research [22,23]. In particular, previous research has emphasized that voluntariness of use is not only a moderating factor but also a key determinant in technology acceptance, highlighting the importance of autonomous choice over mandatory implementation [23]. As technology advances, its adoption is often mandated rather than voluntary, and while mandatory adoption may increase initial uptake in organizational settings, it can ultimately hinder long-term sustainability [23,55]. Particularly when users are excluded from the early stages of technology development, there is a higher likelihood of resistance due to perceptions that the technology undermines their autonomy [56]. Therefore, involving users through participatory design is essential for the successful implementation of new technologies. Previous research suggests that nurse-led implementation of digital health technology in nursing homes can support successful adoption, indicating that environments which ensure professional autonomy and voluntary engagement promote greater acceptance of such technologies [57]. In this context, for the successful adoption of the platform in supportive housing, it is essential to respect the voluntariness of staff and actively involve them through a user-centered design that prioritizes their needs and experiences.

Our result that performance expectancy is a significant predictor of the intention to use is consistent with previous studies [22,26,31,58–60]. Performance expectancy, a core construct of technology acceptance, reflects the belief that using technology will enhance job performance [21]. Specifically, the perception that new technology offers advantages over existing methods serves as a key facilitator of technology adoption [61]. Therefore, for the successful implementation of the platform, it is essential to foster confidence in the staff that the platform will positively impact on their work performance. In residential settings, staff recognize that ICT can support the independence of people with disabilities and participation in disability services, and this perception leads to a positive attitude toward the use of ICT in such environments [62]. However, despite this positive perception, ICT-based health management systems are yet to be widely adopted, making it difficult to share health information and communicating effectively among the staff [3]. The platform proposed in this study has the potential to address these issues. It enables seamless communication and information sharing among the staff and can provide consistent health management for residents, including hospital visit records, medication management, and health monitoring. Thus, to realize this potential, it is essential to provide comprehensive education and training on the functionality and benefits of the platform. For instance, peer mentoring and practical training can enhance the staff’s proficiency and confidence, which in turn may improve perceived usefulness [35]. Such efforts will increase performance expectancy among the staff, serving as a vital factor in promoting their acceptance of the platform. Ultimately, this will ensure the successful integration of the platform into supportive housing environments.

Consistent with previous studies [58,59], this study found that facilitating conditions significantly influenced the intention to use the platform. A systematic review on mHealth adoption identified facilitating conditions as key factors in promoting the acceptance of healthcare-related ICT [61]. In this study, facilitating conditions include organizational and technological environment, as well as technical support. These conditions make it easier for users to access and use new technology [21]. Prior research has found that organizational support significantly influences the adoption of eHealth among healthcare professionals supporting individuals with intellectual disabilities and has emphasized the importance of providing adequate training [16]. However, challenges remain in interoperability and reliability of systems in disability care contexts [63]. This issue is particularly relevant in supportive housing settings, where time and human resources are limited, highlighting the necessity of establishing strategies to create an environment that facilitates platform use. Thus, it is necessary to establish a stable network and provide technical support and training for the staff in supportive housing. Furthermore, in the UTAUT model, facilitating conditions have been shown to significantly affect use behavior [21]. Supporting this, a study employing the UTAUT model underlined the impact of facilitating conditions on the use of new technologies in rehabilitation [31]. Therefore, future research should explore how facilitating conditions influence not only the intention to use but also actual use behavior.

This study found that eHealth literacy significantly influenced the intention to use the platform. Specifically, individuals with higher eHealth literacy had a stronger intention to use the platform. This finding is consistent with previous research indicating that higher eHealth literacy levels increased the likelihood of using patient health information management technologies [39]. eHealth literacy training enhances digital competency and supports positive technology use [64], which suggests the need for educational and training strategies to improve the eHealth literacy of staff. Improving eHealth literacy will enable the staff in supportive housing to better understand and utilize the platform’s functions and the information it offers, ultimately driving the successful adoption of the platform. When developing the platform, it is also crucial to consider design strategies that enable the staff with varying levels of eHealth literacy to effectively use the platform. In particular, the interface should be intuitive and user-friendly, so even users with limited eHealth literacy will perceive that the platform is a useful and accessible tool [39–41].

Although our insignificant effect of social influence and effort expectancy contrasts with most previous studies [22,26,39,50,58,65], it is consistent with the findings from other studies on technology adoption in healthcare settings [16,31]. In care settings for people with disabilities, staff’s intention to use ICT was found to be influenced by its perceived benefits [16,62]. This suggests that the perceived usefulness of the platform in supporting the health management of people with disabilities may play a more significant role than social influence or ease of use. Moreover, in supportive housing, staff primarily work in shifts and provide individual care to residents, which limits opportunities for direct interaction and information exchange among colleagues. This organizational structure may create a context in which social influence operates less strongly. However, these results should be interpreted with caution. It is important to consider that this study was conducted before the platform was fully developed, meaning that users did not have direct experience with the actual usability and functionality of the platform. Future research should investigate the impact of effort expectancy and social influence on platform adoption based on the actual usage experience after the platform has been fully developed.

This study has several limitations. First, the study was conducted with staff in a specific region of South Korea, limiting the generalizability of the findings. Future research should include a broader range of regions and job categories. Second, the staff participating in the survey may have had a preference for such platforms, which could have influenced their willingness to respond. Therefore, the results should be interpreted with caution. Third, since the platform was still in the pre-development stage, the study could not assess actual user experience or evaluate the platform’s design or operational effectiveness. Fourth, this study did not examine the moderating effects of variables such as gender and age, as suggested in the UTAUT model, indicating the need for future research to explore these moderating effects with larger, more diverse samples. Lastly, due to limited information on use behavior and long-term effects following platform implementation, further research is needed to evaluate the long-term use of the platform and its impact on use behavior.

### Theoretical implications

This study contributed to the existing theoretical literature and offered new perspectives in supportive housing for people with disability. First, it extends the original UTAUT model by integrating eHealth literacy as predictors of behavioral intention. Our findings highlight the importance of eHealth literacy in the adoption of the platform within supportive housing. Second, this study contributes to the theoretical development of the UTAUT model by demonstrating its applicability in supportive housing for people with disability, which has been underexplored in previous research. By applying and validating the extended UTAUT model, the study helps bridge a gap in the literature and offers a more comprehensive understanding of technology adoption for health management in supportive housing environments.

### Practical implications

Our findings suggest several practical implications to ensure the successful implementation of the platform in supportive housing for people with disabilities. First, it is essential to encourage the voluntary use of the platform within the organization. Enforcing mandatory use may lead to staff resistance, which could hinder the successful adoption of the platform. Second, systematic education and training for the staff are necessary. This requires not only instruction on how to use the platform, but also education that enhances understanding of its specific benefits in the field. Third, establishing organizational support and a stable technical environment is crucial for the reliable operation of the platform. It is vital to assess the availability of technical resources (such as adequate Wi-Fi and devices) as well as organizational support (such as financial assistance and education) required for platform use in these settings. Lastly, considering the needs and capabilities of the staff is a critical step in the development phase. Supportive housing employs staff members of diverse backgrounds and experiences, leading to differences in technological competence and eHealth literacy. Therefore, it is important to design the platform with a user-centered approach, ensuring an intuitive and accessible interface.

## Conclusion

This study examined the key factors influencing the health information communication and management platform among the staff in supportive housing for people with disabilities, applying the UTAUT model. The findings highlight that voluntariness of use, performance expectancy, facilitating conditions, and eHealth literacy significantly influenced the staff’s intention to use the platform in supportive housing. Based on these findings, fostering voluntary engagement, enhancing performance expectations, ensuring organizational and technical support, and considering users’ eHealth literacy are essential factors for the successful implementation of the platform. This study advances the UTAUT model by applying it to residential setting for people with disabilities and integrating eHealth literacy. Moreover, the results of this study provide a significant contribution by offering valuable foundational data for the implementation and activation of such platforms in supportive housing, as well as practical implications for establishing user-friendly strategies for their future design and development.

## Supporting information

S1 Data Raw data of this study.
(XLSX)
